# Relationship between teacher-student relationship and peer victimization: a multiple mediator model

**DOI:** 10.3389/fpsyg.2026.1801143

**Published:** 2026-04-22

**Authors:** Pingyan Zhou, Jinqi Dong, Jian Liu

**Affiliations:** 1 School of Psychology, Qufu Normal University, Qufu, China; 2 Dongguan High School, Dongguan, Guangdong, China; 3 Collaborative Innovation Centre of Assessment for Basic Education Quality, Beijing Normal University, Beijing, China

**Keywords:** depression, multiple mediator model, peer relationship, peer victimization, teacher-student relationship

## Abstract

**Introduction:**

Growing evidences reveal intimate links between teacher-student relationship (TSR) and peer victimization, yet the underlying mechanisms remain unclear.

**Methods:**

This study examined these associations using data from 65,868 fourth graders (9.56 ± 0.62, 53.60% males) through a multiple mediator model. All participants completed four questionnaires.

**Results:**

This study revealed that TSR was associated with peer victimization exclusively via indirect pathways (the direct effect was non-significant), with three significant mediators: (1) peer relationship (standardized indirect effect = −0.135); (2) depression (−0.040); and (3) the sequential pathway through peer relationship and depression (−0.070). Peer relationship demonstrated the strongest mediating effect and preceded emotion factors, indicating that peer relationship as spillover of skills or behavioral pattern may have more stable and lasting impact on peer victimization than emotion spillover.

**Conclusion:**

These findings suggest that peer victimization is fundamentally connected to peer relationship quality, highlighting the need for relationship-centered interventions. The earlier the intervention for peer victimization occurs, the more effective it is, particularly during the critical period when self-esteem are forming.

## 1 Introduction

Peer victimization intervention is a global challenge, which is defined as repeated, intentional aggressive behavior by peers that involves a real or perceived power imbalance, resulting in physical, verbal, or relational harm to the victim (Olweus, 1993). Victims often find it difficult to escape from the bullying situations (Olweus, 1993). Meanwhile, the proportion of primary and secondary school students worldwide experiencing victimization ranged from 21% (Shojaei et al., 2009) to 60% (Qiu et al., 2024; Xing et al., 2023). Peer victimization has obvious detrimental effects on victims’ physical, psychological, and behavioral outcomes, and adverse effects continue to exist even after the bullying stops. Compared with individuals not being bullied, those experiencing victimization show elevated depression, anxiety, and loneliness, along with reduced self-esteem (Ireland and Qualter, 2008; Wang et al., 2021). Also, being bullied can trigger high**-**risk behaviors in children, such as sleep disorders (van Geel et al., 2016), poor academic performance, and even dropping out (Wei and Williams, 2004). In severe cases, peer victimization increases self-harming behaviors (Bento et al., 2021) and suicidal attempts and behaviors (Gini and Espelage, 2014). Further, students who have been bullied at school are more likely to experience victimization in their future workplaces (Schäfer et al., 2004). Despite existing laws, addressing peer victimization is still difficult. Researchers call for evidence-based solutions and more study of factors like teacher-student relationship (Bjereld et al., 2017). In practice, 73% of teachers report major challenges in handling bullying (Zhang, 2002), highlighting the need to better understand how teacher-student dynamics can help prevent peer victimization.

### 1.1 Teacher-student relationship and peer victimization

One of the primary interpersonal relationships for children at school is teacher-student relationship, which refers to the dyadic relationship characterized by varying degrees of closeness, conflict, and dependency between a student and their teachers (Hamre et al., 2001; Pianta, 1999). It functions as a key mesosystem link, influencing a child’s experience in the classroom and peer group. Supportive teacher-student relationship improves children’s academic adaptation, concentration, and ability to handle social and academic challenges (Hughes and Kwok, 2006). Teachers’ care can directly protect children from being bullied (Fang et al., 2023). A close relationship between children and teachers is related to a reduction in physical attacks children experienced (Troop-Gordon and Kopp, 2011). Also, teacher**-**student relationship quality can predict the likelihood of children being bullied 5 months later (Demol et al., 2020). Children with supportive teacher ties are linked to less bullying, conversely, conflictual ties increase victimization risk (Fang et al., 2023; Marengo et al., 2021). Despite its importance, how teacher-student relationship affects peer victimization remains underexplored in both research and anti-bullying programs, such as the Olweus Bullying Prevention Program. Sulkowski et al. deemed that healthy teacher**-**student relationship has robust negative prediction effects on peer victimization in unexplored ways (Sulkowski and Simmons, 2018), such as through skills or emotion.

### 1.2 The relationship among TSR, peer relationship, and peer victimization

Researchers deemed that integrating teacher-student relationship with peer-level process can strongly strengthen anti-bullying interventions (Bouchard and Smith, 2017; Demol et al., 2020). Studies suggest that supportive teacher-student relationship improves children’s peer interactions through multiple interconnected mechanisms (Bouchard and Smith, 2017). Teachers facilitate group activities and provide social skills training, while the quality of the teacher-student interaction itself serves as a relational template. Through sensitive feedback and support, teachers help shape children’s expectations, core beliefs, and interaction behaviors, which children then carry into their peer relationship (Bouchard and Smith, 2017; Luckner and Pianta, 2011; Troop-Gordon and Kopp, 2011). In this study, peer relationship quality is operationalized as children’s subjective appraisal of their social connectedness and acceptance among peers, assessed via the reverse-scored loneliness scale (Asher et al., 1984). We acknowledge that this measure captures the perceived affective experience of peer relationship (i.e., the distress associated with perceived social isolation) rather than objective indicators of peer acceptance, peer status, or friendship quality. While loneliness is conceptually distinct from these constructs, it is strongly correlated with them in childhood (Asher and Paquette, 2003; Ladd and Ettekal, 2013) and represents a developmentally significant aspect of peer relationship quality—namely, the child’s subjective sense of belonging. High-quality peer relationships provide a sense of belonging and social support. Although Hughes et al. demonstrated that teacher-student relationship is mutually related to peer relationship in a bidirectional manner (Hughes and Chen, 2011), a 5-year longitudinal study revealed that, from a developmental perspective, children’s early relationship with teachers (as non-parental authority figures) are theorized to influence the skills, expectations, and social behaviors they bring to peer contexts (Howes, 2010). Also, Pianta deemed that teacher-student relationship is significantly related to the development of one’s social competence, such as peer relationship (Pianta, 1999). This suggests that TSR may serve as a foundational factor on the quality of children’s peer interactions.

Further, supportive peer relationship reduces the risk of victimization. Godleski et al. (2015) found that children who have peer support are less likely being bullied (Godleski et al., 2015). Peer relationship can mitigate victimization by providing strong social support and fostering group acceptance (Flaspohler et al., 2009), which is in turn negatively related to peer victimization (de Bruyn et al., 2009). Malcolm et al. revealed that strong reciprocal friendships decrease being bullied through enhanced peer support (Malcolm et al., 2006). Also, a longitudinal study found that children with a reciprocal best friend in class experience less victimization a year later, while those without one face an increased likelihood of being bullied (Boulton et al., 1999). Fang et al. (2023) demonstrated that teacher-student relationship primarily is associated with peer victimization through indirect pathways (Fang et al., 2023), both high levels of conflict and dependence in teacher-student relationship correlated with school adjustment problems—including school avoidance, peer aggression, and social withdrawal—which is in turn associated with higher peer victimization through lower peer status (Fang et al., 2023). Hence, we propose Hypothesis 1: Peer relationship would serve as a mediating factor between teacher-student relationship and peer victimization.

### 1.3 The relationship among TSR, depression, and peer victimization

Depression encompasses a state of persistent sadness, loss of interest, and related cognitive and somatic symptoms that impair functioning (Kovacs, 1992). According to self-determination theory, children’s healthy development depends on satisfying three fundamental needs: Interpersonal relatedness, autonomy, and competence (Deci and Ryan, 1985). If children’s needs are not met in reality for long time, it may lead to internalizing problems, such as depression. Teacher support, characterized by encouragement, warmth, and care, meets students’ need for meaningful connection. When teachers are autonomy-supportive, they bolster children’s self-determination and enhance the quality of the teacher-student relationship, with a key protective outcome being the mitigation of depression (Zhang et al., 2022). Also, supportive teacher-student relationship enhances students’ social-cognition, self-worth, and promote a robust sense of social competence (Lim, 1989; Sween, 2023), which can alleviate students’ internalized distress (Lester et al., 2013) and reduce their risk of depression (Chen et al., 2020). Moreover, teachers supporting student autonomy contribute to a reduction in depressive symptoms among children over time (Zhang et al., 2022). Thus, TSR may alleviate depression in children by satisfying their basic needs, which in turn reduces their depression-linked behavioral styles.

Peer victimization can be predicted by depression (Kochel et al., 2012; Kochel and Rafferty, 2020). Each unit increases in depression raised the risk of direct victimization by 20% (Boel-Studt and Renner, 2014). Kochel and colleagues proved that children’s socially helpless behavior served as a significant predictor of their depressive symptom, ultimately leading to subsequent experiences of peer victimization (Kochel and Rafferty, 2020). However, a meta-analysis suggested a bidirectional link between depression and peer victimization, with no difference in correlation coefficients (Christina et al., 2021). Relationships between these variables may vary across age groups and cultures, especially during elementary school—a critical period for self-concept development (Juan et al., 2011). This bidirectional nature underscores the complexity of their relationship. Hence, further research is needed to clarify the direction of this relationship. The current study investigates one theoretically-derived directional pathway within this reciprocal cycle: how depression, as a consequence of interpersonal difficulties, may subsequently increase victimization risk. This perspective is aligned with the interpersonal risk model (Patterson and Capaldi, 1990). Two longitudinal studies have revealed that depression predicted future incidents of peer victimization (Bilsky et al., 2013; Sentse et al., 2017). Then, we propose Hypothesis 2: The association between TSR and peer victimization would operate via depression.

### 1.4 The relationship among TSR, peer relationship, depression, and peer victimization

Both attachment to parents and peers are risk factors for predicting depression, and peer attachment becomes more influential as conflicts with parents growing (Cyranowski et al., 2000; Holmbeck and Hill, 1991; Kullik and Petermann, 2013). Numerous studies have found that depression is negatively related to peer relationship (Franzen et al., 2024; Jiang et al., 2025; Sulkowski and Simmons, 2018). Attachment to peers was negatively related to depression through dysfunctional emotion regulation (Allen et al., 2007; Kullik and Petermann, 2013) and diminished sense of belonging or relatedness to others caused by low-status in peers (Baumeister and Leary, 1995; Slavich and Irwin, 2014). Hence, we theorize that teacher-student relationship might exert an indirect effect on depression through its influence on peer relationship quality. While competing models exist (e.g., the symptoms-driven model) (Nolen-Hoeksema et al., 1992), the present study is conceptually aligned with the interpersonal risk model (Patterson and Capaldi, 1990), which posits that enduring interpersonal difficulties, such as peer rejection or low-quality friendships, constitute a chronic stressor that can lead to the development of depressive symptoms. Supporting this view, longitudinal studies with children in middle childhood have found that peer relationship problems predict increasing in depressive symptoms over time, even after controlling for prior depression (Chen et al., 2012; Jiang and Wang, 2020; Panak and Garber, 1992; Zimmer-Gembeck et al., 2009). Therefore, within the specific context of exploring the origins of peer victimization in the school ecology, we examine the pathway wherein poor peer relationships (associated with TSR) contribute to depression, which then may elevate victimization risk. Thus, we proposed Hypothesis 3: Peer relationship and depression would play a chain-mediating role between TSR and peer victimization.

Currently, the trend of peer victimization is becoming increasingly prevalent among younger students (Xing et al., 2023). The number of primary school students who experience bullying is substantially larger than that of adolescents (Boel-Studt and Renner, 2014). Fourth**-**grade students were at the end of middle childhood and beginning of late childhood. Both stages have a special significance for peer victimization. First, peer victimization peaks during middle childhood (Finkelhor et al., 2013). The need for peer interaction during middle childhood has gradually increased. Children pay more attention to peer relationship and their social status. To maintain these, they often use relational aggression, which fuels relational victimization (Zhang et al., 2009). Second, as peer interactions rise in middle childhood, children begin forming social role identities (Boel-Studt and Renner, 2014). If children are being bullied for a long time, they may develop negative social role identity and behavioral patterns in their peer context, formationing a negative self-concept in late childhood (Juan et al., 2011). Once the social role identity pattern is integrated into a child’s self**-**concept, it could have long**-**term negative effects on their experiences of peer victimization. For example, Schafer et al. found that students who have experienced victimization in primary and secondary schools are more likely to be bullied in their workplace in the future (Schäfer et al., 2004). Thus, targeting fourth-grade students in the current study may have the greatest potential for preventing and intervening in the trajectory of victimization.

### 1.5 An integrated theoretical framework and conceptual definition

This study integrates attachment theory, self-determination theory (SDT), and the interpersonal risk model within a developmental-ecological framework (Bronfenbrenner, 1979). From attachment theory, a supportive teacher-student relationship (TSR) fosters a positive internal working model, shaping children’s expectations and behaviors in peer contexts (Bowlby, 1969). SDT posits that TSR satisfies the need for relatedness, buffering against depression (Deci and Ryan, 1985). The interpersonal risk model further suggests that chronic peer relationship difficulties constitute a stressor that precipitates depressive symptoms (Patterson and Capaldi, 1990). Finally, the spillover hypothesis (Edwards and Rothbard, 2000) explains how skills and emotional patterns transfer from teacher-student to peer systems, culminating in a sequential pathway to victimization.

While extensive evidence confirms a bidirectional, reciprocal relationship between peer victimization and depression (Christina et al., 2021), the primary aim of this study is to elucidate how a distal relational context (TSR) might ultimately contribute to the risk of victimization. We propose that TSR was associated with victimization primarily through its impact on more proximal factors: first by shaping peer relationship quality (a social-behavioral pathway), and subsequently by influencing emotional adjustment (an affective pathway). This sequential model (TSR → Peer relationship → Depression → Victimization) synthesizes the aforementioned theories: TSR satisfies relatedness needs (SDT) and shapes working models (Attachment), which facilitate better peer ties; poor peer ties constitute an interpersonal risk (Interpersonal Risk Model) for depression; and depressive symptoms, through associated social-behavioral deficits (e.g., withdrawal, helplessness), may increase vulnerability to victimization (Kochel and Rafferty, 2020). This represents one plausible pathway within the complex ecology of bullying, acknowledging that victimization can also exacerbate depression and strain all relationships. While we draw on developmental theories (e.g., attachment theory, interpersonal risk model) to propose a theoretically-derived sequential model (TSR → Peer relationship → Depression → Victimization), we acknowledge that the cross-sectional design precludes any causal or temporal inferences. Our model tests the statistical plausibility of this theoretically-specified pattern of associations, but alternative directions (e.g., victimization leading to depression, or reciprocal relationships among all variables) are equally plausible and require longitudinal data for confirmation.

### 1.6 The present study

Guided by the integrated theoretical framework outlined above, the present study aims to examine the specific mechanisms linking teacher-student relationship to peer victimization among fourth-grade students. We posit that TSR was not associated with victimization directly but through intermediary psychosocial processes. Specifically, we test a multiple mediation model to assess two parallel and one sequential indirect pathway: (1) Hypothesis 1: Peer relationship quality will mediate the association between TSR and peer victimization (TSR → Peer relationship → Victimization); (2) Hypothesis 2: Depression will mediate the association between TSR and peer victimization (TSR → Depression → Victimization); (3) Hypothesis 3: Peer relationship quality and depression will sequentially mediate this association in a chain (TSR → Peer relationship → Depression → Victimization).

**Figure 1:**
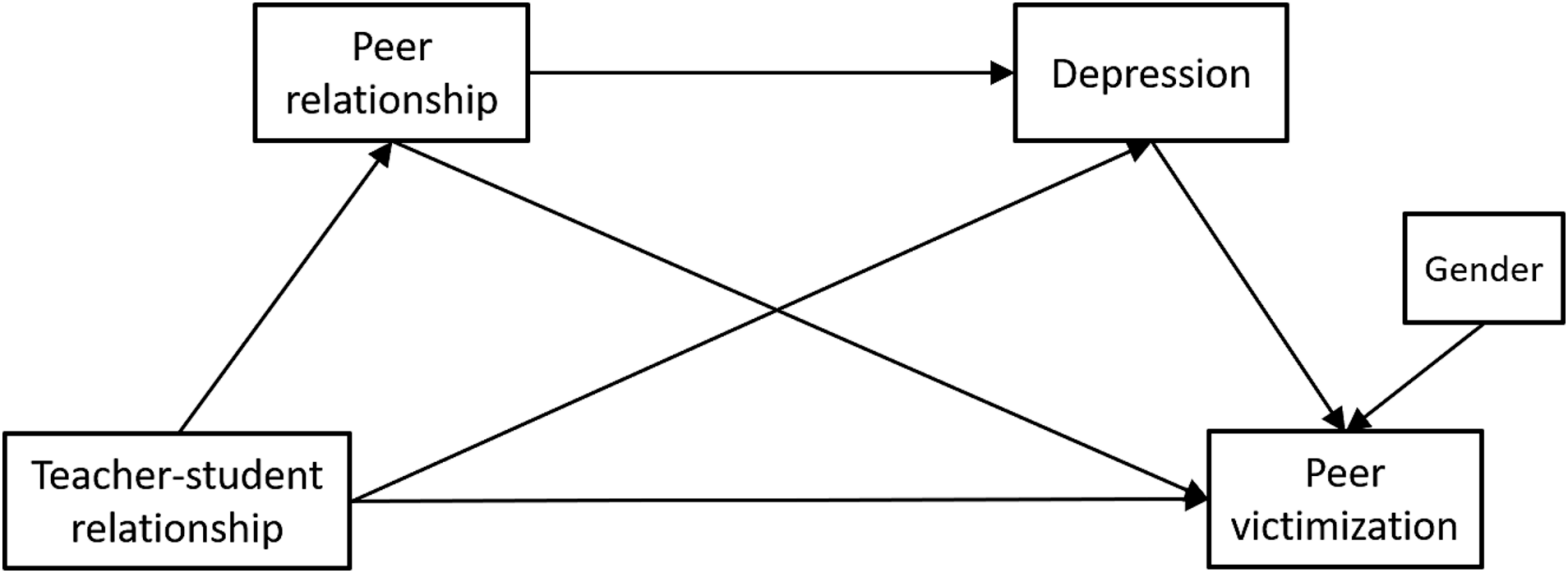
A theoretical model. The direction of the arrows is based on theoretical derivation, but since the data are cross-sectional, it only indicates associations rather than causation.

This model represents a test of the proposition that supportive TSR fosters better peer relationships (satisfying relatedness needs and providing social skills), which in turn protects against depression (reducing interpersonal risk). Lower depression, associated with more adaptive social behaviors, is then hypothesized to decrease vulnerability to victimization. By testing this chain, we seek to clarify how relational experiences at different levels of the school ecology (adult-child, child–child) and emotional adjustment jointly contribute to the risk of peer victimization (Figure 1).

## 2 Materials and methods

### 2.1 Participants

This research employed a cross**-**sectional school-based survey targeting fourth grade students in two Chinese cities during September and October 2015. In Zhengzhou City, all 280 urban primary schools participated, encompassing every fourth**-**grade student. In Shijiazhuang City, a three stages sampling approach were used: (1) Random selection 10 urban districts; (2) Random selection of 50 schools from these districts; and (3) Inclusion of all fourth**-**grade students from selected schools. The initial sample comprised 65,868 participants (53.60% male), after excluding cases with missing data (*N* = 1,819; accounting for 2.76%) and univariate outliers beyond ±3 standard deviations (*N* = 2,260; accounting for 3.43%), the final valid sample comprised 61,789 fourth-grade students. All surveys were administered in classroom settings, after obtaining parental consent to all aspects of the data collection, and teacher-led explanations of study objectives. Students voluntarily completed 6 standard scales under confidential conditions, with uniform protocols across all sites.

### 2.2 Measures

#### 2.2.1 The Revised Peer Relationship Scale

(Reverse-scored as a proxy for Perceived Peer Relationship Quality). The Peer Relationship Scale was a 10-item scale adapted from the original Children’s Loneliness Scale (Asher et al., 1984), which perceived peer relationship quality. We employed the reverse score of reported loneliness as a proxy indicator for the construct of perceived peer relationship quality. This approach was justified based on established theory and empirical evidence: Loneliness was conceptualized as the distressing emotional experience arising from a perceived deficit in the quality or quantity of one’s social relationships (Asher and Paquette, 2003). In child and adolescent populations, loneliness showed strong negative correlations with direct measures of peer acceptance, friendship quality, and social satisfaction (Ladd and Ettekal, 2013; Asher and Paquette, 2003). Thus, while not a direct measure of observed peer interactions, lower loneliness (and its reverse score) served as a valid indicator of a child’s subjective appraisal of their social connectedness and acceptance among peers—a core aspect of peer relationship quality. Participants rated each item on a 4-point Likert scale (1 = definitely matches; 4 = definitely does not match). The total score was the average of the 10 reverse-scored items, with higher scores indicating lower loneliness and, by proxy, better perceived peer relationship quality. In the present study, the Cronbach’s alpha was 0.926. The CFA yielded marginal fit indices, χ^2^_(34)_ = 490.18, *p* ≤ 0.001, CFI = 0.92, TLI = 0.90, SRMR = 0.04, RMSEA = 0.09. While the CFI and TLI approached conventional thresholds, the RMSEA exceeded the recommended cutoff of 0.08 (Hu and Bentler, 1999), suggesting some misspecification in the factor structure.

#### 2.2.2 The Children’s Depression Inventory: Short Form (CDI: S)

The Children’s Depression Inventory: Short form (CDI: S; Kovacs, 1992) was a 10 item self-report measure designed to access depressive symptoms in Children in grades 4 ~ 9 (Kovacs, 1992). Participants rated each item on a 3-point scale (0 ~ 2) based on their feelings over the past 2 weeks. The total score was the average of the scores from the 10 items. Higher scores indicated increased severity. In the current study, the Cronbach’s alpha was 0.822. The results of the CFA showed acceptable fit indices, χ^2^_(34)_ = 225.44, *p* < 0.001, CFI = 0.94, TLI = 0.92, SRMR = 0.03, RMSEA = 0.06.

#### 2.2.3 The Peer Victimization Questionnaire

The Peer Victimization Questionnaire was a 7-item adaptation of the Bully/Victim Questionnaire (Olweus, 1993), designed to assess the frequency and forms of victimization students experienced the previous semester. Participants rated each item on a five-point scale (0 = never, 1 = 1 time, 2 = 2 times, 3 = 3 ~ 4 times, and 4 = more than 5 times), reflecting their recent experiences. The total score (sum of all 7 items) indicated victimization severity, with higher scores denoting greater frequency. The Cronbach’s alpha was 0.790 in this study. The CFA revealed suboptimal fit, χ^2^_(14)_ = 1146.09, *p* ≤ 0.001, CFI = 0.93, TLI = 0.90, SRMR = 0.04, RMSEA = 0.13. The elevated RMSEA indicates potential problems with the factor structure of this adapted measure in our sample.

#### 2.2.4 The Teacher-Student Relationship Questionnaire

The Teacher-Student Relationship Questionnaire was revised from the PISA student questionnaires, which included 5 items rated on a 5-point scale (1 = definitely disagree; 5 = definitely agree). Responses were created by averaging the five items. Higher scores indicated a better perceived teacher-student relationship in children. This study measured the teacher-student relationship between children and their Chinese, Mathematics, and Science teachers, respectively. In this study, the Cronbach’s *α* coefficients of the three scales were 0.880, 0.904, and 0.879 separately. The results of the CFA for teacher-student relationship with the Chinese teacher displayed an acceptable fit, χ^2^_(5)_ = 534.11, *p* < 0.001, CFI = 0.98, TLI = 0.97, SRMR = 0.02, RMSEA = 0.09. CFA indicated acceptable validity of the scale about teacher**-**student relationship with the Mathematics teacher, χ^2^_(5)_ = 603.65, *p* < 0.001, CFI = 0.99, TLI = 0.97, SRMR = 0.02, RMSEA = 0.09. The results of CFA were good and acceptable for the scale of teacher**-**student relationship with the Science teacher, χ^2^_(5)_ = 107.24, *p* < 0.001, CFI = 0.99, TLI = 0.98, SRMR = 0.01, RMSEA = 0.04. In the follow**-**up analysis, three types of teacher-student relationships were treated as observable variables. The latent variable of teacher-student relationship was created by synthesizing the three observable variables using Mplus 7.0, and CFA indicated acceptable fit indices, χ^2^_(14)_ = 127.93, *p* < 0.001, CFI = 0.99, TLI = 0.97, SRMR = 0.02, RMSEA = 0.05.

### 2.3 Data analysis

Analyses were conducted using SPSS 26.0 and Mplus 7.0, with statistical significance set at *p* ≤ 0.05. The analysis proceeded in three stages:

*Stage 1*: Preliminary analyses and data screening. From the initial dataset (*N* = 65,868), we excluded cases with missing data on any study variable (*N* = 1,819; 2.76%) and univariate outliers exceeding ±3 standard deviations (*N* = 2,260; 3.43%), resulting in a final analytic sample of 61,789 (retention rate = 93.81%). Descriptive statistics and bivariate correlations. We computed means, standard deviations, and Pearson correlations among all study variables. Correlation effect sizes were interpreted using Cohen’s guidelines (Cohen, 1988): small (*r* = 0.10), medium (*r* = 0.30), and large (*r* = 0.50).

*Stage 2*: Confirmatory factor analysis (CFA). We conducted CFAs for each scale to verify the factor structure. Model fit was evaluated using the comparative fit index (CFI; acceptable ≥ 0.90), Tucker-Lewis index (TLI; acceptable ≥ 0.90), root mean square error of approximation (RMSEA; acceptable ≤ 0.08), and standardized root mean square residual (SRMR; acceptable ≤ 0.08), in accordance with guidelines (Hu and Bentler, 1999).

*Stage 3*: Multiple mediation analysis. Controlling for gender, we tested a multiple mediation model in Mplus 7.0 using maximum likelihood estimation with robust standard errors (MLR). The model examined: (a) the direct path from TSR to peer victimization; (b) the indirect path through peer relationship (H1); (c) the indirect path through depression (H2); and (d) the sequential indirect path through peer relationship and depression (H3). Statistical significance of indirect effects was assessed using bias-corrected bootstrap confidence intervals (5,000 resamples) (Preacher and Hayes, 2008). Effects were considered significant if the 95% confidence interval did not include zero.

## 3 Results

### 3.1 Descriptive analyses

Table 1 presented the means, standard deviations, and correlations among the 7 variables. The three teacher-student relationship measures (Chinese, Mathematics, and Science) showed negative correlations with peer victimization and depression, but positive correlations with peer relationship and gender. Peer victimization was positively associated with depression, but negatively linked with peer relationship and gender. Depression exhibited negative correlations with peer relationship and gender, while peer relationship was positively related to gender. Independent-sample t-tests revealed significant gender differences (*p* < 0.05) for 6 variables. Consequently, subsequent analysis included gender as a covariate.

**Table 1:** Descriptive statistics and correlations between five variables.

Variables	1	2	3	4	5	6	7
Gender	1						
Peer victimization	−0.15**	1					
TSR-C	0.07**	−0.19**	1				
TSR-M	0.01	−0.20**	0.51**	1			
TSR-S	−0.03**	−0.16**	0.47**	0.54**	1		
Peer relationship	0.12**	−0.49**	0.36**	0.34**	0.27**	1	
Depression	−0.07**	0.46**	−0.29**	−0.30**	−0.23**	−0.61**	1
Means	1.46	0.87	4.05	3.95	3.84	3.28	3.20
Standard deviation	0.50	0.92	0.838	0.904	0.99	0.54	3.11

**p < 0.01; TSR-C, Teacher-student relationship with the Chinese teacher; TSR-M, Teacher-student relationship with the Mathematics teacher; TSR-S, Teacher-student relationship with the Science teacher.

According to Cohen’s guidelines for effect sizes (Cohen, 1988), correlations of 0.10, 0.30, and 0.50 are considered small, medium, and large, respectively. Peer victimization showed a large positive correlation with depression (*r* = 0.48 *p* ≤ 0.001) and a large negative correlation with peer relationship (*r* = −0.49, *p* ≤ 0.001). TSR measures showed small to medium negative correlations with peer victimization (rs ranging from −0.16 to −0.20) and depression (rs from −0.23 to −0.30), and medium positive correlations with peer relationship (rs from 0.27–0.36).

### 3.2 Testing for the multiple mediated effects

The mediation analysis proceeded in four steps. First, we examined the total effect of TSR on peer victimization without mediators, which was significant (*c* = −0.253, *p* ≤ 0.001). Second, we added the mediators to assess direct and indirect effects. Third, we used bias-corrected bootstrapping (5,000 samples) to generate 95% confidence intervals for indirect effects (Preacher and Hayes, 2008). Fourth, we calculated the proportion mediated as the indirect effect divided by the total effect, although we acknowledge this metric should be interpreted cautiously, especially when the direct effect is not significant (Preacher and Kelley, 2011).

**Figure 2:**
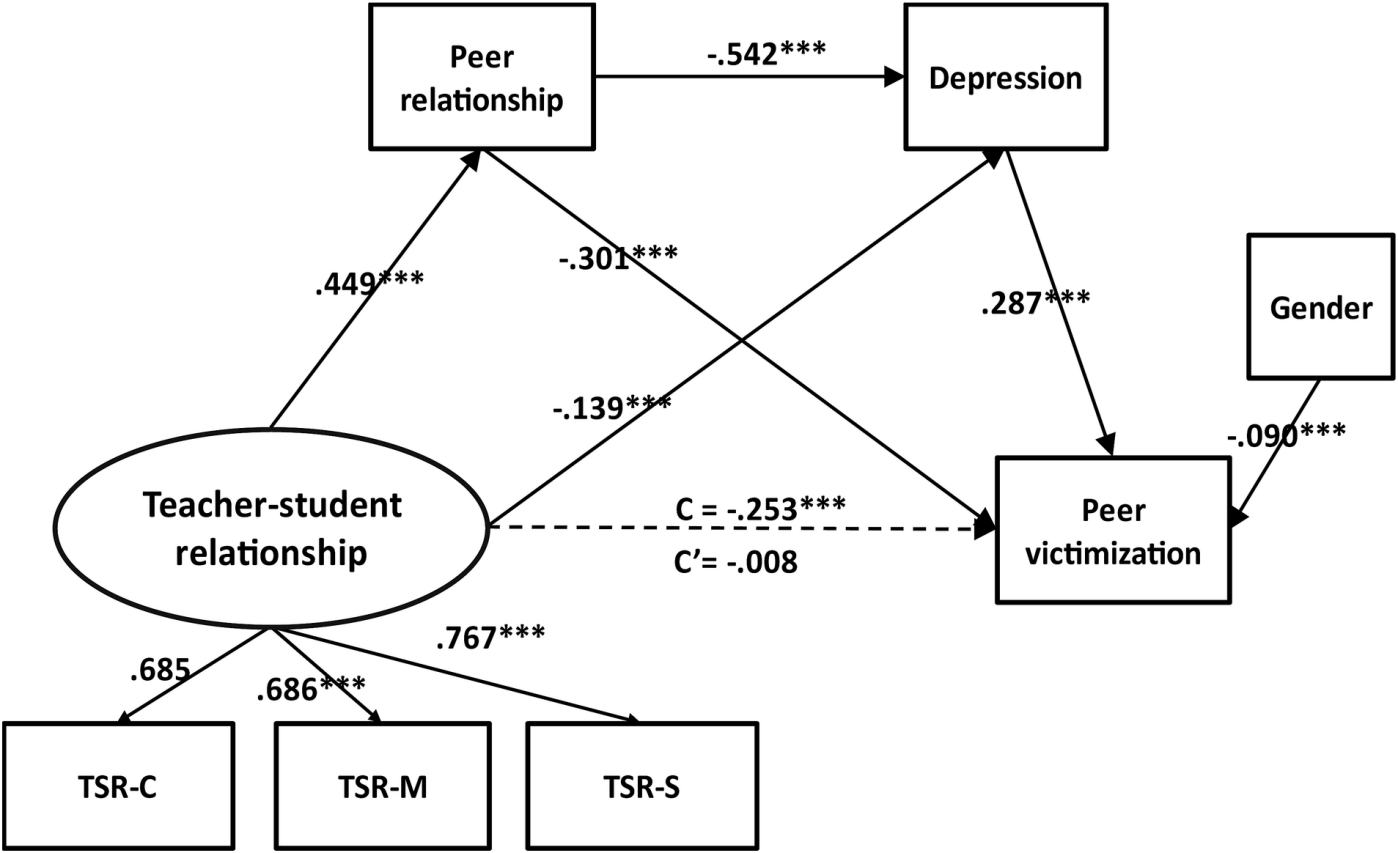
A multiple mediation model of the association between teacher-student relationship and peer victimization via peer relationship and depression. Standardized regression coefficients and proportion mediated were provided along the paths (***p < 0.001). The proposed directional paths are based on theoretical frameworks (e.g., the interpersonal risk model, the spillover theory) and specific longitudinal evidence supporting segments of the chain. However, the cross-sectional data used in this study can only test associations, not causal directions. Alternative pathways (e.g., victimization → depression) were also theoretically plausible and require longitudinal examination.

The bootstrapping (Samples = 5,000) method was conducted to verify the chain of peer relationship and depression mediated the association between teacher**-**student relationship and peer victimization. The 95% confidence intervals (95% CI) that did not contain zero indicated effects that were significant. With the original data set (*N* = 61, 789), results showed good model fit [χ^2^_(11)_ = 201.16, *p* ≤ 0.001, CFI = 0.98, TLI = 0.96, RMSEA = 0.06]. Indirect effects, and effect size (proportion mediated) were displayed in Table 2. In the multiple mediation model (Figure 2), the direct effect of teacher**-**student relationship on peer victimization was not significant, *c*’ = −0.008, *p* > 0.05. Teacher**-**student relationship was positively associated with peer relationship, *β* = 0.449, *p* < 0.001. Peer relationship was negatively related to peer victimization, *β* = −0.301, *p* < 0.001. Peer relationship fully mediated the association of teacher**-**student relationship and peer victimization. Teacher**-**student relationship was negatively associated with depression, *β* = −0.139, *p* < 0.001. Depression was positively related to peer victimization, *β* = 0.287, *p* < 0.001. Depression also served as a complete mediator. Further, peer relationship was negatively correlated with depression, *β* = −0.542, *p* < 0.001. Thus, the chain mediation of teacher-student relationship—peer relationship—depression—peer victimization was significant. The worse the teacher**-**student relationship, the worse the peer relationship, the more prone to depression, and the higher the risk of peer victimization.

**Table 2:** Bootstrap analyses of the magnitude and statistical significance of indirect effect.

Model pathways	β (estimate)	Effect size	Ratio of total indirect effect	95% CI
Direct effect	−0.008			−0.026, 0.003
Total indirect effect	−0.245	−0.245/−0.253 = 96.838%		−0.250, −0.239
TSR → peer relationship → peer victimization	0.449*−0.301 = −0.135	−0.135/−0.253 = 53.359%	−0.135/−0.245 = 55.102%	−0.140, −0.130
TSR → depression peer → victimization	−0.139*0.287 = −0.040	−0.040/−0.253 = 15.811%	−0.040/−0.245 = 16.327%	−0.043, −0.037
TSR → peer relationship → depression → peer victimization	0.449*−0.542*0.287 = −0.070	−0.070/−0.253 = 27.668%	−0.070/−0.245 = 28.571%	−0.073, −0.067

Standard errors and test coefficients are values for standardized estimates. TSR, teacher-student relationship.

The ratio of the indirect effect divided by the total effect of teacher-student relationship and peer victimization, *c* = −0.253, *p* < 0.001. The 95% CI for the mediating effect of peer relationship between teacher-student relationship and peer victimization was (−0.140, −0.130), representing a moderate-to-large indirect effect (standardized *β* = −0.135). The 95% CI for the mediating effect of depression was (−0.043, −0.037), indicating a small but significant indirect effect (standardized *β* = −0.040). The 95% CI for the chain mediating effect of peer relationship and depression was (−0.073, −0.067), reflecting a small-to-moderate indirect effect (standardized *β* = −0.070). All three indirect effects were statistically significant, with the combined indirect effects accounting for the majority of the total association between TSR and peer victimization.

## 4 Discussion

The current study investigated the intermediary mechanism between teacher-student relationship and peer victimization. We found a negative effect on peer victimization from teacher-student relationship, which is consistent with existing research findings (Bouchard and Smith, 2017; Demol et al., 2020). Students with a healthy teacher-student relationship tend to receive timely and effective support and assistance from their teachers when facing victimization (Shin and Ryan, 2017). These relationships provide students with social skills support and academic guidance, thereby reducing their likelihood of being bullied. In contrast, conflicts between teachers and students can be positively related to peer victimization (Marengo et al., 2021). There are two possible reasons. First, due to teachers’ authority, students may avoid building relationships with children who have conflicts with their teachers (Bouchard and Smith, 2017). Second, children may be unable to obtain support from teachers, and are unlikely to resist or retaliate when being bullied, making them targets for bullying.

Our results also found that teacher-student relationship was significantly related to peer victimization only indirectly through the two full mediators of peer relationship and depression. First, peer relationship fully explained the pathway from TSR to peer victimization, supporting Hypothesis 1, indicating that peer relationship is a crucial factor in the relationship between the two main variables. Previous research suggested that teacher-student relationship shapes peer relationship through multiple ways (Bouchard and Smith, 2017): (1) positive teacher-student relationship can provide children with a sense of security and belonging, helping them develop healthy peer relationship; (2) teachers’ social skills training is beneficial for children to resolve conflicts and contradictions among peers; and (3) peer relationship can be shaped by teacher-student relationship via social referrals. Peers may observe the interaction between the teacher and the child and then judge whether they would establish peer relationship with that child. Hence, peer’s attitudes toward a child can be affected by the relationship between the child and their teacher. Compared to those who are rejected by both teachers and peers, more positive interactions between teachers and individuals who are only rejected by peers can help reduce being bullied (Taylor, 1989). More recent studies have also documented that positive teacher-student relationship can buffer against peer rejection and promote prosocial behavior, thereby reducing victimization risk (Fang et al., 2023; Gao et al., 2022). Further, to reduce the risk of being ostracized by a group, bullies often target children with poor peer relationship (Salmivalli, 2010). If children form mutual friendship at an early stage, they are less likely to be bullied, even if this friendship breaks down later (Boulton et al., 1999). The higher the popularity, the greater the influence among peers, and the less likely they are to be bullied (de Bruyn et al., 2009). Positive peer relationship represents peer support and higher status within the peer group, which have deterrent effects on bullies and can reduce the likelihood of being bullied. Taken together, these findings suggest that peer victimization is closely intertwined with peer relationship quality, indicating that addressing peer dynamics should be a central component of anti-bullying interventions.

Second, depression fully mediates the effect of teacher-student relationship on peer victimization. This is consistent with the existing literature and the Hypothesis 3. Researchers believe that teacher support can reduce depressive risk (Lester et al., 2013). The interpersonal risk model of depression emphasizes the crucial role of interpersonal relationship on depression (Patterson and Capaldi, 1990). For example, the persistent stress associated with poor interpersonal relationship, which is often resistant to short-term intervention, significantly increases vulnerability to depression (Kochel et al., 2012). Depression contributed to victimization vulnerability in several ways: to begin with, social deficits—such as complaining, criticism, or social anxiety—and maladaptive relationship choices attract bullies and increase victimization risk (Reijntjes et al., 2010). Furthermore, they may exhibit negative behavioral patterns such as aggression and destructiveness, which may provoke peer attacks (Tzeletopoulou et al., 2018). Finally, due to depressive symptom, victims tend to respond to bullying with withdrawal, passivity, and fear rather than counterattacks or retaliation (Zhou et al., 2003). This does not facilitate the resolution of the problem, but instead invites further bullying, resulted in a vicious cycle that is difficult to change. Researches further specify that depressive symptoms in children are linked to social information processing biases (e.g., hostile attribution) and reduced assertiveness, making them less effective in deflecting bullying attempts (Fang et al., 2023; Keenan et al., 2010; Rantanen et al., 2021). Hence, social behavior and relationship handling deficiencies caused by depression make children more likely to be bullied.

Third, the current study found that peer relationship and depression not only have separate mediating effects on the relationship between teacher-student relationship and peer victimization, but also demonstrated a chain mediating effect. The spillover theory hypothesizes that individuals are embedded in multiple interdependent social systems, and that experiences in one system may be related to functions in another (Kaufman et al., 2020). Edwards and Rothbard categorize spillover into domains such as skills, behaviors, and emotions (Edwards and Rothbard, 2000). Applied to our context, this suggests that the relational patterns and social competencies developed within teacher-student interactions may transfer (spill over) to the peer domain, potentially influencing the quality of peer relationships. Subsequently, the affective states (e.g., depression) associated with peer relationship quality maybe further associated with risk for victimization. This theoretical framework supports the proposed sequence in our model, wherein teacher-student relationship is linked to peer victimization via peer relationship and then depression.

The findings from this large-scale study offer robust support for an integrated theoretical model linking teacher-student relationship to peer victimization via peer relationship quality and depression. Our results extend previous research in several key theoretical ways: (1) the strongest mediating pathway through peer relationship (*β* = −0.135) strongly supports the spillover of relational schemas and social competencies from the teacher-student dyad to the peer group (Edwards and Rothbard, 2000). This aligns with attachment theory’s premise that the internal working model, initially shaped by primary caregivers and later modified by teachers, guides expectations and behaviors in other relationships (Bouchard and Smith, 2017; Bowlby, 1969). Unlike transient emotional states, these relational schemas involve relatively stable skills, behavioral patterns, and expectations (Song, 2016), which may explain their larger and more stable mediating effect. This finding underscores that victimization is fundamentally a relational problem rooted in a child’s position within the peer social ecology, which is itself influenced by their relationship with authority figures; (2) the significant sequential mediation (TSR → Peer Relationship → Depression → Victimization; *β* = −0.070) provides empirical validation for the interpersonal risk model of depression within a school context (Patterson and Capaldi, 1990). It demonstrates how chronic interpersonal adversity in the form of poor peer relationship can foster depressive symptoms, which then, through associated social-behavioral deficits like withdrawal or social helplessness (Kochel and Rafferty, 2020), increases vulnerability to victimization. This chain elucidates one specific mechanism through which the frequently observed bidirectional link between depression and victimization may be initiated from the relational environment (Christina et al., 2021); (3) the minimal direct effect of TSR on victimization highlights the predominantly indirect nature of this association. This reinforces an ecological perspective (Bronfenbrenner, 1979), suggesting that influences from one system (teacher-student) on an outcome in another system (peer victimization) are largely channeled through proximal processes in the intervening system (peer relationship and individual affect).

It demonstrates how chronic interpersonal adversity in the form of poor peer relationship can foster depressive symptoms, which then, through associated social-behavioral deficits like withdrawal or social helplessness (Kochel and Rafferty, 2020), increases vulnerability to victimization. This chain elucidates one specific mechanism through which the frequently observed bidirectional link between depression and victimization may be initiated from the relational environment (Christina et al., 2021); (3) the minimal direct effect of TSR on victimization highlights the predominantly indirect nature of this association. This reinforces an ecological perspective (Bronfenbrenner, 1979), suggesting that influences from one system (teacher-student) on an outcome in another system (peer victimization) are largely channeled through proximal processes in the intervening system (peer relationship and individual affect).

The current study revealed teacher-student relationship, peer relationship, and depressive mood as risk factors for peer victimization among school children, suggested that intervention for victimization should not only focus on individual factors, but also should attach great importance to teacher-student (Poling et al., 2022) and peer relationships (Dai et al., 2022). Specially, early childhood represents a critical period for cultivating supportive teacher-child and peer interactions, as positive relational schemas during this formative stage may serve as protective factors against subsequent peer victimization. Early intervention in childhood, as studied here, is critical to prevent the consolidation of negative relational schemas and emotional patterns. Hence, these findings advocate for a relationship-centered, multi-systemic approach to bullying prevention in early childhood. Interventions should move beyond focusing solely on the bully-victim dyad and actively strengthen both teacher-student and peer relationship qualities. School-based programs could train teachers in practices that build closeness and reduce conflict (e.g., banking time) (Poling et al., 2022) while simultaneously implementing peer-led initiatives that foster inclusive classroom climates and social–emotional skills (Dai et al., 2022).

This study had several limitations. First, the data in the current study all came from students’ self-report, and there may be a common method bias in the collected data. In future research, incorporating multi-informant reports (teachers, peers, and parents) and social network analysis could provide a more objective and nuanced picture of peer dynamics. Second, cross-sectional studies cannot establish temporal precedence among variables. Future longitudinal or experimental designs are needed to verify the temporal precedence of these pathways and to examine potential bidirectional effects. Third, all the participants were fourth-grade students. However, peer victimization can exist throughout primary school stage. Selecting only fourth-grade students might slightly weaken the representativeness across all elementary school grades. In future research, sampling from all primary school grades could be employed to enhance the representativeness of the sample. Fourth, the measurement model fit indices for some scales, particularly peer victimization (RMSEA = 0.13), were suboptimal. Although large samples can inflate χ^2^ statistics and sometimes produce acceptable fit by some standards, the RMSEA value for peer victimization exceeded conventional thresholds for acceptable fit (Hu and Bentler, 1999). This suggests potential issues with the factor structure of the adapted Olweus scale in this Chinese sample of fourth graders. Future research should employ psychometrically stronger measures or utilize multi-informant approaches (e.g., peer nominations, teacher reports) to assess victimization more reliably. Fifth, while we controlled for gender, other potentially confounding variables such as family socioeconomic status (SES) and prior victimization history were not available in this dataset. Their omission limits the causal inferences that can be drawn. Sixth, the operationalization of peer relationship quality via reverse-scored loneliness is a significant conceptual limitation. Loneliness captures subjective emotional distress arising from perceived social isolation, rather than objective relational positions such as peer acceptance, peer status, or friendship quality (Asher and Paquette, 2003; Maes et al., 2019). While loneliness is correlated with these constructs, it is conceptually distinct and may artificially inflate the observed associations with depression, given the emotional overlap between loneliness and depressive symptoms. This may have strengthened the TSR → peer relationship (loneliness) → depression pathway in our model. Future research should employ multi-dimensional measures of peer relationships, including peer nominations for acceptance/rejection, friendship quality scales, and social network analysis, to disentangle the unique and joint contributions of these different aspects of peer relations.

In conclusion, this study explored the mechanism of teacher-student relationship on peer victimization, and verified the mediating effects of peer relationship and depression. In practice, interventions should emphasize the development of children’s adaptive skills and emotion regulatory capabilities to strengthen their interpersonal coordination abilities in various relationships, especially peer relationship. The earlier the intervention, the better the outcome.

## Data Availability

The raw data supporting the conclusions of this article will be made available by the authors, without undue reservation.
